# Genome sequence of *Shewanella corallii* strain A687 isolated from pufferfish (*Sphoeroides spengleri*)

**DOI:** 10.1590/1678-4685-GMB-2018-0314

**Published:** 2020-02-17

**Authors:** Gustavo P.R. Azevedo, Pedro H. C da Paz, Hannah K. Mattsson, Ana Paula B. Moreira, Luciana Leomil, Gabriela Calegário, Luciana Appolinario, Lívia Vidal, Bruno S. Silva, Luciane A. Chimetto Tonon, Diogo A. Tschoeke, Gizele D. Garcia, Fabiano L. Thompson, Cristiane C. Thompson

**Affiliations:** 1 Universidade Federal do Rio de Janeiro, Institute of Biology and SAGE-COPPE, Rio de Janeiro, RJ, Brazil.; 2 Universidade de São Paulo, Instituto de Química de São Carlos (IQSC-USP), São Carlos, SP, Brazil.; 3 Universidade Federal do Rio de Janeiro, Departamento de Ensino de Graduação, Campus UFRJ – Macaé Professor Aloisio Teixeira, Macaé, RJ, Brazil.

**Keywords:** Shewanella, gammaproteobacteria, genome, gene prediction, secondary metabolism

## Abstract

We present here the genome sequence of *Shewanella corallii* strain A687 isolated from pufferfish *Sphoeroides spengleri* (Family Tetraodontidae). The assembly consists of 5,215,037 bp and contains 284 contigs, with a G+C content of 50.3%.

The genus *Shewanella* comprises 67 recognized species. These bacteria are Gram-negative, rod-shaped, facultatively anaerobic gammaproteobacteria and frequently isolated from marine environments (MacDonell and Colwell, 1985; Sugimoto *et al.*, 2018). *Shewanella* species are involved in the production of antimicrobial metabolites and tetrodotoxin, a strong neurotoxin (Matsui *et al.*, 1989; Simidu *et al.*, 1990; Magarlamov *et al.*, 2017). *Shewanella corallii* was recorded from red sea coral (Shnit-Orland, 2010). The aim of the present study was to determine the genome sequence of *Shewanella corallii* strain A687.

*S. corallii* A687 was isolated from pufferfish *Sphoeroides spengleri* (Family Tetraodontidae), in Arraial do Cabo (Brazil) in 2016. Genomic DNA was extracted according to Pitcher’s protocol (Pitcher *et al.*, 1989) and used for 300-bp paired-end library preparation with Nextera XT DNA Sample Preparation Kit. The genome was sequenced using MiSeq (Illumina, San Diego, CA, USA) as previously described (Walter *et al.*, 2016). Sequences obtained were pre-processed using PRINSEQ software to remove reads smaller than 35 bp and low-score sequences (Phred 30) (Schmieder and Edwards, 2011). Sequence reads were assembled using A5-Miseq (Coil *et al.*, 2015) and CAP3 software (Huang and Madan, 1999). The gene prediction and functional annotation were performed using the RAST server (Overbeek *et al.*, 2014). Secondary metabolism was analyzed by antiSMASH (Weber *et al.*, 2015) and clustered regularly interspaced short palindromic repeat (CRISPR) arrays were determined with CRISPRFinder (Grissa *et al.*, 2007).

The sequencing generated a total of 4,557,272 reads and 768,079,097 bp that were assembled in 284 contigs (N50=298,540 bp). The estimated genome size is 5,215,037 bp with G+C of 50.3%, and a coverage of 146-fold. RAST predicted 4,555 coding sequences, and 175 RNA sequences (147 tRNAs, 11 16S rRNAs, 6 23S rRNAs, and 11 5S rRNAs). Analyzing the genes predicted by RAST, a total of 74 genes were involved in resistance to antibiotics and toxic compounds including copper homeostasis (N=8); bile hydrolysis (N=2); cobalt-zinc-cadmium resistance (N=20); resistance to fluoroquinolones (N=4); arsenic resistance (N=4); copper homeostasis: copper tolerance (N=6); tetracycline resistance, ribosome protection type II (N=2); beta-lactamase (N=5); multidrug resistance efflux pumps (N=22); resistance to chromium compounds (N=1); 24 genes for the metabolism of aromatic compounds, including salicylate ester, chloroaromatic and quinate degradation. Phage elements sequences (N=44) were found in this genome, and CRISPRs arrays candidates were predicted in four sequences. We searched through subsystems for genes associated to symbiosis. Genes encoding type I, (lapBCDE, lapL, lapP, RTX and TolC) and type II (TadA, TadB, TadC, VirB11, RcpC, CpaB and CpaF) secretion systems were detected. We also identified 16 genes related to vitamin B12 synthesis and four LuxR gene families (Bondarev *et al.*, 2013).

## Data availability

This whole-genome shotgun project has been deposited in GenBank under the accession number QRCS00000000. The version described in this paper is the first version.
